# Supplementary material for: Multi-decade biomass dynamics in an old-growth hemlock-northern hardwood forest, Michigan, USA
Source: PeerJ. 2014 Sep 30;2:e598. doi: 10.7717/peerj.598 (PMC4183956; doi:10.7717/peerj.598)
Supplement: Table S1 [file peerj-02-598-s001.docx]

**Supplemental Table 1**

Sources for allometric equations used for estimation of aboveground biomass of living trees and contribution to CWD input at death. All equations are based on diameter at breast height. Where necessary, totals were otained by estimating components separately and summing. See text for discussion of criteria for selection of eqations.

*References Cited, Supplemental Table 1*

Chapman, J. W., & Gower, S. T. 1991. *Aboveground production and canopy dynamics in sugar maple and red oak trees in southwestern Wisconsin*. Canadian Journal of Forest Research 21(10):1533–1543.

Crow, T. R., & Erdmann, G. G. 1983. *Weight and volume equations and tables for red maple in the Lake States.* Research Paper NC-242. St. Paul, MN: US Dept. of Agriculture, Forest Service, North Central Forest Experiment Station.

Hocker, H. W., & Early, D. J. 1983. *Biomass and leaf area equations for northern forest species* (Research Paper No. 102). New Hampshire Agricultural Experiment Station, University of New Hampshire.

Jenkins, J. C., Chojnacky, D. C., Heath, L. S., & Birdsey, R. A. 2003. *National-scale biomass estimators for United States tree species*. Forest Science 49(1):12–35.

Koerper, G. J., & Richardson, C. J. 1980. *Biomass and net annual primary production regressions for Populusgrandidentata on three sites in northern lower Michigan*. Canadian Journal of Forest Research 10(1):92–101.

Morrison, I. K. 1990. *Organic matter and mineral distribution in an old-growth Acersaccharum forest near the northern limit of its range*. Canadian Journal of Forest Research 20(9):1332–1342.

Parker, G. R., & Schneider, G. 1975. *Biomass and Productivity of an Alder Swamp in Northern Michigan*. Canadian Journal of Forest Research 5(3):403–409.

Perala, D. A., & Alban, D. 1994. *Allometric biomass estimators for aspen-dominated ecosystems in the upper Great Lakes.* (Research Paper No. NC-314). USDA Forest Service, North Central Forest Experiment Station.

Schmitt, M. D. C., & Grigal, D. F. 1981. *Generalized biomass estimation equations for Betulapapyrifera Marsh.* Canadian Journal of Forest Research 11(4):837–840.

Ter-Mikaelian, M. T., & Korzukhin, M. D. 1997. *Biomass equations for sixty-five North American tree species*. Forest Ecology and Management 97(1):1–24.

Young, H. E., Ribe, J. H., & Wainwright, K. 1980. *Weight tables for tree and shrub species in Maine*. Maine. Life Sciences and Agriculture Experiment Station. Miscellaneous report.
