# Supplementary material for: Multi-decade biomass dynamics in an old-growth hemlock-northern hardwood forest, Michigan, USA
Source: PeerJ. 2014 Sep 30;2:e598. doi: 10.7717/peerj.598 (PMC4183956; doi:10.7717/peerj.598)
Supplement: Table S2 [file peerj-02-598-s002.docx]

**Supplemental Table 2: Biomass density by plot and species**

Biomass density in Mg/ha for all species and plots. Values are given for three sample dates: the earliest (1962), most recent (2009), and 1989. Plot groups are as discussed in text.

**Supplemental Table 2 (cont.)**
