# Supplementary material for: Multi-decade biomass dynamics in an old-growth hemlock-northern hardwood forest, Michigan, USA
Source: PeerJ. 2014 Sep 30;2:e598. doi: 10.7717/peerj.598 (PMC4183956; doi:10.7717/peerj.598)
Supplement: Table S3 [file peerj-02-598-s003.docx]

Supplemental Table 3:

Coarse woody debris in 2007, by species and decay class. All values are in Mg/ha

Supplemental Table 3 (cont.)
